# Natural language processing to automate a web-based model of care and modernize skin cancer multidisciplinary team meetings

**DOI:** 10.1093/bjs/znad347

**Published:** 2024-01-10

**Authors:** Stephen R Ali, Thomas D Dobbs, Adib Tarafdar, Huw Strafford, Beata Fonferko-Shadrach, Arron S Lacey, William Owen Pickrell, Hayley A Hutchings, Iain S Whitaker

**Affiliations:** Reconstructive Surgery and Regenerative Medicine Research Centre, Institute of Life Sciences, Swansea University Medical School, Swansea, UK; Welsh Centre for Burns and Plastic Surgery, Morriston Hospital, Swansea, UK; Reconstructive Surgery and Regenerative Medicine Research Centre, Institute of Life Sciences, Swansea University Medical School, Swansea, UK; Welsh Centre for Burns and Plastic Surgery, Morriston Hospital, Swansea, UK; Reconstructive Surgery and Regenerative Medicine Research Centre, Institute of Life Sciences, Swansea University Medical School, Swansea, UK; Welsh Centre for Burns and Plastic Surgery, Morriston Hospital, Swansea, UK; Neurology and Molecular Neuroscience Group, Institute of Life Science, Swansea University Medical School, Swansea University, Swansea, UK; Health Data Research UK, Data Science Building, Swansea University Medical School, Swansea University, Swansea, UK; Neurology and Molecular Neuroscience Group, Institute of Life Science, Swansea University Medical School, Swansea University, Swansea, UK; Health Data Research UK, Data Science Building, Swansea University Medical School, Swansea University, Swansea, UK; Neurology and Molecular Neuroscience Group, Institute of Life Science, Swansea University Medical School, Swansea University, Swansea, UK; Health Data Research UK, Data Science Building, Swansea University Medical School, Swansea University, Swansea, UK; Neurology and Molecular Neuroscience Group, Institute of Life Science, Swansea University Medical School, Swansea University, Swansea, UK; Department of Neurology, Morriston Hospital, Swansea, UK; Faculty of Medicine, Health and Life Science, Swansea University Medical School, Swansea, UK; Reconstructive Surgery and Regenerative Medicine Research Centre, Institute of Life Sciences, Swansea University Medical School, Swansea, UK; Welsh Centre for Burns and Plastic Surgery, Morriston Hospital, Swansea, UK

## Abstract

**Background:**

Cancer multidisciplinary team (MDT) meetings are under intense pressure to reform given the rapidly rising incidence of cancer and national mandates for protocolized streaming of cases. The aim of this study was to validate a natural language processing (NLP)-based web platform to automate evidence-based MDT decisions for skin cancer with basal cell carcinoma as a use case.

**Methods:**

A novel and validated NLP information extraction model was used to extract perioperative tumour and surgical factors from histopathology reports. A web application with a bespoke application programming interface used data from this model to provide an automated clinical decision support system, mapped to national guidelines and generating a patient letter to communicate ongoing management. Performance was assessed against retrospectively derived recommendations by two independent and blinded expert clinicians.

**Results:**

There were 893 patients (1045 lesions) used to internally validate the model. High accuracy was observed when compared against human predictions, with an overall value of 0.92. Across all classifiers the virtual skin MDT was highly specific (0.96), while sensitivity was lower (0.72).

**Conclusion:**

This study demonstrates the feasibility of a fully automated, virtual, web-based service model to host the skin MDT with good system performance. This platform could be used to support clinical decision-making during MDTs as ‘human in the loop’ approach to aid protocolized streaming. Future prospective studies are needed to validate the model in tumour types where guidelines are more complex.

## Teaser

What’s already known about this topic?

- Cancer multidisciplinary team (MDT) meetings are under intense pressure to reform given the rapidly rising incidence of skin cancer and the national mandate for protocolized streaming.
- There is not enough time to discuss complex patients, attendance is not optimal, the right information is often not used to inform discussions and MDTs are unable to fulfil their secondary roles in data validation, audit and education.

What does this study add?

- It is feasible that this platform could be used to support clinical decision making during the skin MDT as ‘human in the loop’ approach to aid protocolized streaming.
- This could aid management decisions for ‘simple’ cases and free up time to discuss more ‘complex’ patients.

## Introduction

Multidisciplinary team meetings (MDTs) are an integral component in the management of skin cancer. There is mounting evidence, however, to support reform in how MDTs operate. The skin MDT has been shown to be costly when compared to other specialties as well as poorly attended, with only 26 per cent quorate by membership and 69 per cent quorate by meeting frequency^1^. With an estimated 50 000 shortfall in NHS clinical staff in England reported as of 2021, this is unlikely to improve^2^. The skin MDT is also unique in that the incidence of skin cancer is rising faster than other malignancy^3,4^. Furthermore, the remit of the skin MDT is expanding. Historically, UK guidelines have recommended that all cases of high-risk squamous cell carcinoma and malignant melanoma were discussed at the specialist skin cancer multidisciplinary team (SSMDT), but omitted any recommendations on referral for basal cell carcinoma (BCC)^5–7^. This has now changed, with the most up-to-date British Association of Dermatologists (BAD) UK BCC guidelines highlighting the pivotal role of the MDT in the management of high-risk BCC. Given these new recommendations, caseloads of local skin multidisciplinary teams (LSMDTs) and SSMDTs are only set to rise. Urgent solutions are therefore required to address the skin MDT workload. The skin cancer community has additionally identified a number of potential areas for MDT improvement^8^. One specific area was the mandate for protocolized streaming at a national level, with guidance on streamlining according to clinical complexity issued by NHS England and NHS Improvement in 2020^9^.

Innovative solutions to these problems are needed. Since the coronavirus disease 2019 (COVID-19) pandemic the concept of virtual MDTs has expanded, with many moving to an online teleconferencing format. In a recent study, this was shown to maintain or improve standards in the domains of: communication, chairing and decision-making; training, clinical trials recruitment and audit; as well as data security and patient confidentiality, when compared to a face-to-face MDT^10^. Attendance would also likely improve by allowing members to be involved without having to travel between sites for meetings.

The effectiveness of the virtual MDT could be further enhanced by the inclusion of novel technologies. This could reduce the burden on skin cancer MDTs by facilitating the protocolization of treatment pathways and supporting management decisions for ‘simple’ cases. The nascent fields of data science, artificial intelligence (AI) and natural language processing (NLP) all represent huge opportunities. NLP can be defined as a set of techniques used to convert written text into interpretable data sets through either rule-based or machine learning models^11^. Using NLP to extract surgical outcomes from electronic health records (EHR) is accelerating across disciplines and clinical outcomes research^12^, with an aim to improve outcomes, increase safety and aid service planning. The Reconstructive Surgery & Regenerative Medicine Research Centre has previously developed and validated an automated clinical text extraction system that accurately extracts pathological data from BCC histopathology reports^13^. Moreover, by using this platform, the feasibility of generalizing a validated NLP pipeline to new data within a web application framework was demonstrated^14^.

The primary aim of this study was, by using BCC as a use case, to validate an NLP-based platform to automate evidence-based decisions in a clinical decision support system capable of multiclass classification that align with national skin cancer treatment guidelines.

## Methods

### Study design

A multicentre (Morriston Hospital, Singleton Hospital and Neath Port Talbot Hospital, Wales, UK), pan-specialty, consecutive retrospective analysis of patients with a diagnosis of BCC over a 6-month period from 1 March 2021 to 20 September 2021 was undertaken. Lesions were examined by a consultant histopathologist using the bread loafing cross-section technique^15^.

### Patient identification, inclusion and exclusion criteria

Patients were retrospectively identified from InterSystems TrakCare Lab Laboratory Information Management System (InterSystems TrakCare Lab, Cambridge, Massachusetts, USA), using SNOMED reference term (RT) codes for BCC. SNOMED is a logic-based healthcare terminology used in an EHR. It is a consistent vocabulary for recording patient clinical information^16^. All those patients with a SNOMED RT code for BCC during the study period who were managed by surgery (either non-definitive diagnostic sampling biopsy (punch biopsy, incision biopsy, shave biopsy or curettage) or surgical excision using a predetermined margin) were included. Comma separated variable (CSV) text files were generated from the respective canonical subheadings of the histopathology report.

### General framework

**Fig. 1:**
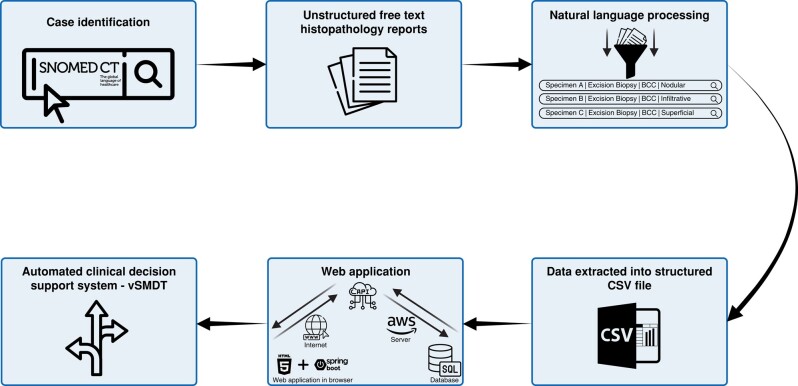
Schematic representation of the virtual skin MDT

The virtual skin MDT (vSMDT) consists of two main parts:; the extraction of the right text from the pathology report using the NLP algorithm and then producing the right recommendation within a web application given the data-extracted histopathology report (*Fig. 1*).

### NLP algorithm

A detailed description and validation of the NLP algorithm has previously been published^13^. A summary of the data extracted from free-text histopathology reports by the pipeline is shown in *Table S1*. In brief, the general architecture for text engineering (GATE) framework was used to build an NLP information extraction system using rule-based techniques. This was validated on previously unseen, de-identified and pseudonymized BCC histopathological reports at the same institution as the current study. The mean precision, recall and F1 score were 86.0 per cent (95 per cent confidence c.i., 75.1–96.9), 84.2 per cent (95 per cent c.i. 72.8–96.1) and 84.5 per cent (95 per cent c.i. 73.0–95.1), respectively. The overall performance of this pipeline is good, and most importantly, compares well with gold standard clinician review (15.5 per cent *versus* 7.9 per cent).

### Web application

Java^TM^ Spring Boot (VMware Incorporated, Palo Alto, California, USA) was used to develop a web application hosted on Amazon Web Services (Amazon.com Incorporated, Seattle, Washington, USA) in EC2. Respective CSV files generated from the NLP pipeline were imported into a relational database management system (RDMS). MySQL Workbench and MySQL Community Server (Oracle Corporation, Austin, Texas, USA) were used as the platform for the RDMS. Within the application framework, an application programming interface (API) was developed to automate a clinical decision support system mapped to and adapted from the recommendations for BCC management in adults following primary treatment in the 2021 BAD guidelines (*Table 1*)^7^. A letter template is then generated ready to be sent to the patient, which communicates the diagnosis(es) and provides recommendations for the next course of action. The letter includes a BAD patient information sheet explaining the diagnosis, as well as a Melanoma UK patient information sheet on self-examination. The outcome from the clinical decision support system model was binary (that is, 1/0) rather than using probabilities of the predicted class. A training set of 100 histopathology reports was used to develop the API with 100 per cent accuracy to ensure that correct predictions were made given the data input prior to validation.

**Table 1:** Virtual skin MDT recommendations for histopathology report outcome following primary surgical treatment

Histopathology report outcome	Recommendation
Completely excised single BCC	No follow-up
Incompletely excised BCC lesion (peripheral margin, deep margin or both)	Offer re-excision. If declined follow-up 6 monthly for 2 years
Multiple BCCs	Follow-up 6 monthly for 5 years
Recurrent BCC	Follow-up 6 monthly for 5 years
Supplemental BCC peripheral margin = positive	Offer re-excision. If declined follow-up 6-monthly for 2 years
Supplemental BCC peripheral margin = negative	No-follow-up
Supplemental BCC deep margin = positive	Offer re-excision. If declined follow-up 6-monthly for 2 years
Supplemental BCC deep margin = negative	No follow-up
Punch biopsy, incision biopsy, shave biopsy or curettage AND cancer type = BCC	Further excisional surgery, destructive surgical or non-surgical technique recommended to obtain oncological clearance
Any margin outcome (complete/incomplete) for a benign lesion or non-specific	No follow-up
If cancer type = any other cancer, other in situ or other intermediate	Other cancerous, *in situ* or intermediate lesion. Review of histopathology free text required to guide management

### Variables

Perioperative tumour factors (primary *versus* recurrent) and surgical factors (excision type, diagnosis and margin status) were recorded.

### Outcomes

The primary endpoint was histological margin status, defined as either clear (>0 mm) or involved (0 mm) in line with current Royal College of Pathologists histopathological reporting standards for primary BCC^17^.

### Statistical analysis

Validation was undertaken retrospectively by two independent and blinded expert clinicians acting as ‘the face-to-face MDT’. These clinicians decided on the management outcome after surgery for each patient based on guidelines from the BAD and those used at the authors’ hospital skin MDT (*Table 1*). This was taken as the gold standard to which the vSMDT decision tool performance was compared. Disagreements in management outcomes were resolved by case discussion until a consensus was reached. Cohen’s kappa was used to assess interobserver agreement and ensure consistency of decision-making between cases. Statistical analysis was undertaken in R version 4.1.1 (R Core Team, R Foundation for Statistical Computing, Vienna, Austria). Sensitivity, specificity, positive predictive value (PPV) and negative predictive value (NPV) were calculated to evaluate the performance of the multiclass clinical decision support system. The overall system performance was summarized using the Yardstick package in R. Micro- and macro-averaging are two ways to combine the results of multiple binary classification models into a single multiclass classification model^18^. In micro-averaging, the metric is calculated for each individual class and then averaged across all classes. In macro-averaging, the metric is calculated for each individual class and then the unweighted average is taken across all classes. This can be problematic in imbalanced data sets, because it can result in the model being overly influenced by the majority class. Given the likelihood for class distribution imbalance a micro-averaging approach was adopted to ensure that each class is given equal consideration in the final model. A one-*versus*-rest method was used to evaluate each individual model within the multiclassifier by comparing each class against all the others at the same time using the Classification And REgression Training (CARET) package in R.

An *a priori* sample size calculation was undertaken. Forty cases and 760 controls were required on the assumption of a sensitivity of 0.95, significance level of 0.95, desired precision (delta) of 0.10 and prevalence of 0.05. Controls could be other patients not meeting the singular reference value being tested at that time. Thus, controls can rotate with case patients when the other reference values are tested. A disease prevalence of 0.05 was assumed for the reference values, based on previous work that established the proportion of incomplete excision in the same study population as 5.5 per cent^14^. It was deemed that there would be a similar prevalence amongst the reference values. A complete-case analysis approach was used to handle any missing data. *P* < 0.05 was deemed statistically significant.

## Results

There were 893 patients (1045 lesions) who had a SNOMED RT diagnosis of BCC in their histopathology report. The mean patient age was 74 years (26–102). From the 1045 observations, there were only 10 disagreements, yielding an agreement rate of 99.23 per cent between the two independent clinicians. Cohen’s Kappa coefficient, computed assuming an equal probability of random agreement for the two outcomes, was found to be 0.99, indicating a very high level of agreement that significantly surpasses chance. These results highlight the quality and reliability of the reference standard.

There were 745 instances where the reference group and prediction matched, with the no follow-up criteria being met (*Table 2*). This demonstrated a strong concordance between the vSMDT and human decision-making processes in certain scenarios. When considering the entire data set, the vSMDT showed promising performance. The overall performance of producing the same recommendation as a clinician given the histopathology report was good with an accuracy of 0.92. These results imply that the vSMDT was highly capable of correctly interpreting and making decisions based on the histopathology reports across the vast majority of cases. Overall summary statistics are shown in *Table 3a* and provide an in-depth overview of these performance metrics. Upon further examination, it was observed that the performance of the vSMDT varied depending on the specific type of recommendation being issued. *Table 3b–f* presents a detailed breakdown of the vSMDT performance per recommendation type.

**Table 2:** Baseline confusion matrix of reference (experienced clinician) and prediction recommendations (vSMDT)

	Reference				
Prediction	Follow-up 6-monthly for 5 years	Further excisional surgery, destructive surgical or non-surgical technique recommended to obtain oncological clearance	No follow-up	Offer re-excision. If declined follow-up 6-monthly for 2 years	Other cancerous, *in situ* or intermediate lesion. Review of histopathology free text required to guide management
Follow-up 6-monthly for 5 years	138	7	12	11	3
Further excisional surgery, destructive surgical or non-surgical technique recommended to obtain oncological clearance	0	17	1	1	0
No follow-up	8	7	745	20	4
Offer re-excision. If declined follow-up 6-monthly for 2 years	0	0	5	12	0
Other cancerous, *in situ* or intermediate lesion. Review of histopathology free text required to guide management	0	0	7	1	46

**Table 3:** Performance of (a) overall and (b–f) individual recommendations by the vSMDT

	Prediction					
Statistics	(a) Overall*	(b) Follow-up 6-monthly for 5 years	(c) Further excisional surgery, destructive surgical or non-surgical technique recommended to obtain oncological clearance	(d) No follow-up	(e) Offer re-excision. If declined follow-up 6-monthly for 2 years	(f) Other cancerous, *in situ* or intermediate lesion. Review of histopathology free text required to guide management
Accuracy	0.92	0.99	0.99	0.98	0.97	0.99
Sensitivity	0.92	0.95	0.55	0.97	0.27	0.87
Specificity	1.00	1.00	1.00	1.00	1.00	1.00
PPV	1.00	1.00	1.00	1.00	1.00	1.00
NPV	0.98	0.99	0.99	0.92	0.97	0.99

NPV, negative predictive value; PPV, positive predictive value. *Overall performance calculated by micro-averaging pairwise comparisons.

The vSMDT demonstrated high specificity across all recommendation categories, implying that the issued recommendations were typically correct. Specificity values stood at 1.00 across all categories (*Table 3b–f*), revealing a strong performance in avoiding false positives. On the other hand, the sensitivity that is reflecting the ability to correctly identify cases necessitating a specific recommendation was lower. Notably, two recommendations stood out with clearly lower sensitivity scores. The recommendation for ‘*further excisional surgery, destructive surgical or non-surgical technique recommended to obtain oncological clearance*’ exhibited a sensitivity of 0.55 (*Table 3c*). Similarly, the recommendation ‘*offer re-excision. If declined follow-up every 6 month for 2 years*’ had a sensitivity score of 0.27 (*Table 3e*). Interestingly, of the 22 patients who met the 2021 BAD guideline recommendation of ‘*following discussion at an MDT, offer further standard surgical re-excision to adults with excised high-risk BCC with involved histological margin unless there is a contraindication*’, only 22.7 per cent were actually discussed at an LSMDT or SSMDT. This finding exposes potential gaps in the application of guideline recommendations in the real-world clinical context, underscoring the potential utility of the vSMDT in supporting clinical decision-making.

## Discussion

This study validates a fully automated, virtual, web-based service model to host the skin MDT. High specificity, as demonstrated here, would make the system suitable to be used as a ‘diagnostic test’ in the vSMDT context. Highly specific tests are used for ruling in a disease, as it rarely misclassifies those without a disease, which is desirable from a clinical decision support system. Given the scope for potential downstream clinical error with even the highest specificity, the vSMDT lends itself to being an adjunct to MDT decision-making and facilitates protocolized streaming with a ‘human in the loop’ as opposed to a fully autonomous system. This aligns with the Topol Review ‘Preparing the healthcare workforce to deliver the digital future’, which anticipates that genomics, digital medicine, AI and robotics will not replace but enhance healthcare professionals, thereby giving them more time to patient care^19^.

Arguably one of the most high-profile use cases of AI as a method of automation in healthcare is the international validation of an AI system for breast cancer screening^20^. McKinney *et al.* found that they were able to develop a system capable of surpassing human experts in breast cancer prediction. In their simulation, the AI system participated in the double-reading process that is used and found a non-inferior performance and reduction of second reader workload by 88 per cent. The NICE Medtech innovation briefing on ‘Artificial intelligence in mammography’ recognizes the value of how AI technologies may improve performance and save time in interpreting mammograms^21^. NHS trusts are beginning to adopt this technology with the AI algorithm Transpara (ScreenPoint Medical, Nijmegen, Netherlands), which uses deep-learning convolutional neural networks, feature classifiers and image analysis algorithms ^21^. At present the NHS Breast Screening Programme (BSP) uses a system of two readers and arbitration to interpret mammograms^22^. BPS is facing a shortage of qualified radiologists. AI technologies in this setting would reduce workloads by replacing one of the two readers, or by performing triage according to the likelihood of an image being malignant. It could also be used to automatically classify images showing a low likelihood of malignancy as normal, and remove these from the images to be reviewed. Similar to the use of AI mammography in breast screening, the vSMDT could deliver comparable benefits and efficiency savings for skin cancer services. In particular, vSMDT-mediated protocolized streaming of ‘low-risk’ cases could take place in advance of the main face-to-face skin MDT. This would go some way to address the issues identified by the Cancer Research UK report into ‘Improving the Effectiveness of Multidisciplinary Team Meetings in Cancer Services’. This report demonstrated that there is not enough time to discuss complex patients, attendance is not optimal, the right information is often not used to inform discussions and that MDTs are unable to fulfil their secondary roles in data validation, audit and education.

Current ‘black-box’ AI models may offer superior performance but model outcomes are not easily explained, causing some to question their suitability for use in high-stakes scenarios such as medicine^23^. The more transparent, ‘glass-box’ nature of explainable AI helps clinicians and patients understand and trust the behaviour of the model. It more easily allows for debugging and improvements to model performance. In this study a rule-based method was used to develop the NLP algorithm. This has the benefit of the rules contained in the model being precise and easy to customize, often simple to implement while being interpretable and explainable. These factors make rule-based methods eminently suitable to the biomedical domain. Rules can, however, become incredibly complex, order matters and maintenance is complicated. Rules are unsuitable for some error types—for example, semantic errors—and require language specific knowledge. Despite these limitations, the performance of the rule-based NLP pipeline was maintained when an API was designed to use these data to predict treatment pathways.

By using a highly specific model like the vSMDT, one would usually expect a trade-off with a lower sensitivity. However, two rules in the model displayed markedly lower sensitivity compared to the other three. In the NLP model that underpins the data extraction of the vSMDT there were 80 separate gazetteers and 445 Java Annotation Patterns Engine (JAPE) rule files in total. Isolating the specific data-extraction error that gets incorrectly transduced into the web application and subsequently contributes to poor sensitivity is likely to be the best strategy to increase performance in this area. The initial NLP pipeline was designed with the aim of improving the quality of routinely collected data for research as well as supporting the vSMDT. A scaled-back NLP pipeline with a smaller number of rules designed to extract the minimum amount of data needed by the vSMDT to make a clinical recommendation would simplify ‘explainability’ and improve the transparency of the decision-making model. General approaches to increasing NLP pipeline performance by increasing the volume and quality of training data are also valid here. The API produced a binary outcome in the clinical recommendation outputted. Instead, using probabilities of the predicted class on a scale, for example 0–100, may be a more nuanced approach and would allow clinicians to define an optimal cut-point value using receiver operating characteristic (ROC) curve analysis.

An alternative strategy to extracting data, converting them into a structured database and creating a series of rules that use such data to predict MDT recommendations (binary or scale) could use machine learning (ML) to directly predict clinical decision pathway(s) from free-text histopathology reports, for example, text classification. This approach could also be easily combined with historical data to improve predictions. A rapidly evolving area of NLP is the evolution of deep-learning models that can be used for this purpose. The two particular types of structure of neural networks used in deep learning are convolutional neural networks (CNNs) and recurrent neural networks (RNNs), with the latter being used for analysis of sequential data such as text. Medical research output in the field of deep learning has predominantly focused on RNNs in imaging rather than CNNs and text analysis, where the images themselves are more easily de-identified and able to be shared as a public data set for use among medical researchers around the world. This is at present probably the most significant barrier to creating high-quality, large-volume training data sets that are necessary for creating and deploying deep learning models. A rapidly evolving area of research that has the potential to change this is the transformer. A transformer represents a new type of AI language model that does not use the traditional methods of RNNs or CNNs^24^. Instead, it uses attention mechanisms, which is a different way of processing information. This makes the transformer simpler and more efficient than other models, and it can be trained faster with fewer resources. Other ML algorithms used in skin cancer MDTs have not been able to demonstrate superior performance in comparison to rule-based techniques. This is illustrated by Andrew *et al.*, who developed a supervised ML algorithm utilizing a decision-tree model trained on a routinely collected SSMDT data set from a single institution to predict MDT decisions for Mohs micrographic surgery *versus* conventional surgery or radiotherapy^25^. Their model was only able to triage 45.1 per cent of patients to a treatment plan.

The authors acknowledge that the external generalizability of this clinical decision support system to other tumour types beyond BCC remains a limitation. Within more complex tumour types, however, there exists a subset of cases that are simpler and could be effectively managed with a protocolized approach. Such a clinical decision support system could play a crucial role in standardizing treatment recommendations for simpler cases, allowing the MDTs to focus complex cases that require more nuanced discussion and decision-making. The quality of histopathological reporting can also significantly impact the external generalizability of the clinical decision support system. As highlighted by Barrett *et al.*, compliance with minimum data set reporting in non-melanoma skin cancer tends to be lower compared to melanoma, indicating potential variances in reporting structure^26^. Paradoxically, there could be better performance when the tool is applied to other tumour types with perceived higher morbidity and mortality, as complex cases often warrant more thorough histopathological reporting, providing more consistent and comprehensive data.

While the vSMDT demonstrates significant potential for improving efficiency in diagnosing and treating BCC, it has certain limitations. A key restriction is its current dependence on the information contained in histopathology reports or EHRs. This means the system’s efficacy is linked to the comprehensive nature and detail within these reports. In its current iteration, the system may not fully consider the wider clinical context of a patient, particularly previous diagnoses like melanoma. The decision-making algorithm does not yet comprehensively integrate prior medical history to influence recommendations. For instance, even in low-risk lesions, patients with a history of melanoma may require continued surveillance and should not be prematurely discharged. Moving forward, the sophistication and utility of the system can be enhanced by incorporating algorithms that can parse through and learn from a broader range of data sources. This would include information on a patient’s prior diagnoses and other relevant clinical details. By doing so, the system will offer more individualized and contextually appropriate recommendations.

Clinical decision support systems, especially if classified as medical devices, must adhere to stringent regulations and standards. According to the UK Medical Device Regulations 2002 (UK MDR 2002), a medical device is defined as ‘*any instrument, apparatus, appliance, software, material or other article, whether used alone or in combination, together with any accessories, including the software intended by its manufacturer to be used specifically for diagnosis or therapeutic purposes or both and necessary for its proper application* …’. If a clinical decision support system aligns with these criteria, it must comply with the regulations outlined by the UK Medicines and Healthcare products Regulatory Agency (MHRA)^27^. The path to compliance starts at conceptualisation, involving the identification of the appropriate legislation applying to the device. This process demands careful consideration and thorough documentation, encompassing details such as product specifications, evidence of safety and effectiveness, results of clinical trials, and information about the manufacturing process. Subsequently, the medical device requires an assessment, the nature of which hinges on the associated risk level. Low-risk devices (class 1) can opt for self-certification, while higher-risk devices (class 2a, 2b and 3) necessitate an evaluation by an MHRA-approved body^28^. The vSMDT with human involvement would likely be classified as a class 1 device. Class 1 devices typically include non-invasive devices like a clinical decision support system, which support rather than dictate clinical decisions. This assessment guarantees that the device’s benefits outweigh the minimized risks, with a successful evaluation culminating in the assignment of a UKCA mark, denoting compliance with UK MDR 2002. Integral to this process is the implementation of a quality management system (QMS)^28^. Legally required for medical device development, a QMS delineates processes minimising the production, deployment and surveillance risks of medical devices. A robust QMS presents structure for essential company processes revolving around device safety and efficacy. The QMS should be certified to a recognized standard like ISO 13485, which ensures comprehensive document management, risk assessment, sign-off procedures and decision records.

While the vSMDT has shown promise in this initial retrospective assessment it is important to note that these findings should be considered preliminary and hypothesis-generating. The next essential step towards establishing the validity of this tool would be to apply it prospectively in an independent cohort, allowing for real-time evaluation of its performance and reliability. This will provide a robust confirmation of these initial findings and could have significant implications for practice. Moreover, patient and public involvement is an established part of medical research study design. While the focus of the present study was on validation, a future aspiration is to include patient choice into the recommendations. By co-referencing clinic letters, the NLP algorithm will endeavour to factor in this critical facet to aid decision-making after primary treatment.

## Supplementary Material

znad347_Supplementary_Data

## Data Availability

All data were anonymized prior to collection. There is no data-sharing agreement for the data. Efforts are being made to obtain patient consent and produce a minimum data set for cross-platform testing.

## Reporting standards

Transparent Reporting of a multivariable prediction model for Individual Prognosis or Diagnosis (TRIPOD).
