# Supplementary material for: Natural language processing to automate a web-based model of care and modernize skin cancer multidisciplinary team meetings
Source: Br J Surg. 2024 Jan 10;111(1):znad347. doi: 10.1093/bjs/znad347 (PMC10782209; doi:10.1093/bjs/znad347)
Supplement: znad347_Supplementary_Data [file znad347_supplementary_data.docx]

**Natural language processing to automate a web-based model of care and modernise the skin cancer multidisciplinary team meeting**

Stephen R Ali ^1, 2^, Thomas D Dobbs ^1, 2^, Adib Tarafdar ^1, 2^, Huw Strafford ^3, 4^, Beata Fonferko-Shadrach ^3,4^, Arron S Lacey ^3, 4^, William Owen Pickrell ^3, 5^, Hayley A Hutchings ^6^, Iain S Whitaker ^1, 2^

1. Reconstructive Surgery and Regenerative Medicine Research Centre. Institute of Life Sciences, Swansea University Medical School, Swansea, UK
2. Welsh Centre for Burns and Plastic Surgery, Morriston Hospital, Swansea, UK
3. Neurology and Molecular Neuroscience Group, Institute of Life Science, Swansea University Medical School, Swansea University, Swansea, UK
4. Health Data Research UK, Data Science Building, Swansea University Medical School, Swansea University, Swansea, UK
5. Department of Neurology, Morriston Hospital, Swansea, UK
6. Swansea University Medical School, Faculty of Medicine, Health and Life Science, Swansea, UK

**Corresponding author:**

Mr Stephen Ali BM MMedSc(Hons) PGCME MAcadMEd FHEA MRCS(Eng)

Reconstructive Surgery & Regenerative Medicine Research Centre, Institute of Life Sciences, Swansea University Medical School, Swansea SA2 8PP, United Kingdom

Tel: 01792205678

Email: stephenrahemali@gmail.com

ORCID: https://orcid.org/0000-0002-9917-3432

**Supplementary Materials - Index**

| **Supplementary Figures and Tables** |  |
| --- | --- |
| **Supplementary Table 1:** Summary of extracted features from BCC histopathology reports. | *page 2* |
| **References** | *page 6* |

**Supplementary Figures and Tables**

**Supplementary Table 1:** Summary of extracted features from BCC histopathology reports.

| **Entity** | **Feature** |
| --- | --- |
| **Accession number** | Value |
| **Excision date** | Day date |
|  | Month date |
|  | Year date |
| **Clinical details** | Tag |
|  | Pre-op diagnosis |
|  | Pre-op diagnosis 2 |
|  | Pre-op diagnosis class |
|  | Pre-op diagnosis class 2 |
|  | Excision type |
|  | Excision type2 |
|  | Excision nature |
|  | Biopsy proven |
|  | Ulcerated |
|  | Body part |
|  | Body part2 |
|  | Multiple scalp |
|  | Lateralization |
|  | Upper or lower |
|  | Anterior or posterior |
|  | Proximal or distal |
|  | Medial or lateral |
|  | Peripheral margin value |
|  | Peripheral margin unit |
|  | Supplemental peripheral margin clock range |
|  | Supplemental peripheral margin clock range 2 |
|  | Deep margin |
|  | Supplemental deep margin clock range |
|  | Supplemental deep margin clock range 2 |
| **Macroscopic details** | Tag |
|  | Pre-op diagnosis |
|  | Pre-op diagnosis 2 |
|  | Ulcerated |
|  | Excision type |
|  | Excision type 2 |
|  | Excision nature |
|  | Body part |
|  | Body part2 |
|  | Multiple scalp |
|  | Lateralization |
|  | Upper or lower |
|  | Anterior or posterior |
|  | Proximal or distal |
|  | Medial or lateral |
|  | 3D specimen size |
|  | 3D specimen size 2 |
|  | 3D specimen size 3 |
|  | 3D specimen size 4 |
|  | Specimen measurement unit |
|  | Max macro tumour diameter |
|  | Max macro measurement unit |
| **Microscopic details** | Tag |
|  | Excision type |
|  | Excision type 2 |
|  | Cancer type |
|  | Cancer type2 |
|  | BCC class |
|  | BCC class 2 |
|  | BCC class 3 |
|  | BCC class 4 |
|  | Differentiation |
|  | Differentiation 2 |
|  | Ulcerated |
|  | Lymphovascular invasion |
|  | Perineural invasion |
|  | Excision completed |
|  | Re-excision outcome |
|  | Recurrent outcome |
|  | Level of invasion |
|  | Clark level |
|  | Stage |
|  | Peripheral clear |
|  | Peripheral clear but close |
|  | Peripheral close |
|  | Supplemental peripheral |
|  | Deep clear |
|  | Deep clear but close |
|  | Deep close |
|  | Supplemental deep |
|  | Frozen section outcome |
| **Microscopic measurements** | Tag |
|  | Measurement type |
|  | Measurement value |
|  | Measurement unit |
|  | Peripheral clock position |
|  | Peripheral clock position 2 |
|  | Peripheral clock position 3 |
|  | Peripheral clock position 4 |
|  | Deep clock position |
|  | Deep clock position 2 |
|  | Deep clock position 3 |
|  | Deep clock position 4 |
| **Requestor** | Clinician name |
|  | Speciality of clinician |
| **Report Details** | Day date |
|  | Month date |
|  | Year date |
|  | Pathologist |
| **Supplementary report** | Tag |
|  | Cancer type |
|  | Cancer type2 |
|  | BCC class |
|  | BCC class 2 |
|  | BCC class 3 |
|  | BCC class 4 |
|  | Differentiation |
|  | Differentiation 2 |
|  | Ulcerated |
|  | Lymphovascular invasion |
|  | Perineural invasion |
|  | Excision completed |
|  | Re-excision outcome |
|  | Recurrent outcome |
|  | Level of invasion |
|  | Clark level |
|  | Stage |
|  | Supplemental peripheral margin |
|  | Supplemental deep margin |
|  | Peripheral clear |
|  | Peripheral clear but close |
|  | Peripheral close |
|  | Deep clear |
|  | Deep clear but close |
|  | Deep close |
|  | Measurement Type |
|  | Measurement Value |
|  | Measurement Unit |
|  | Peripheral clock position |
|  | Peripheral clock position 2 |
|  | Peripheral clock position 3 |
|  | Peripheral clock position 4 |
|  | Deep clock position |
|  | Deep clock position 2 |
|  | Deep clock position 3 |
|  | Deep clock position 4 |
|  | Day date |
|  | Month date |
|  | Year date |
|  | Pathologist |

**References**

1. Ali SR, Dobbs TD, Hutchings HA, Whitaker IS. Composition, Quoracy and Cost of Specialist Skin Cancer Multidisciplinary Team Meetings in the United Kingdom. J Plast Reconstr Aesthet Surg. 2021 Dec;74(12):3335–40.

2. British Medical Association. NHS medical staffing data analysis. Available from: https://www.bma.org.uk/advice-and-support/nhs-delivery-and-workforce/workforce/nhs-medical-staffing-data-analysis (last accessed 3 April 2023)

3. Cancer Research UK. Melanoma skin cancer statistics. Available from: https://www.cancerresearchuk.org/health-professional/cancer-statistics/statistics-by-cancer-type/melanoma-skin-cancer (last accessed 3 April 2023)

4. Cancer Research UK. Non-melanoma skin cancer statistics. Available from: https://www.cancerresearchuk.org/health-professional/cancer-statistics/statistics-by-cancer-type/non-melanoma-skin-cancer (last accessed 3 April 2023)

5. Keohane SG, Botting J, Budny PG, et al. British Association of Dermatologists guidelines for the management of people with cutaneous squamous cell carcinoma 2020. Br J Dermatol. 2021 Mar;184(3):401–14.

6. National Institute for Health and Care Excellence. Melanoma: Assessment and Management (NICE Guideline NG14). National Institute for Health and Care Excellence (NICE) London, UK; 2015.

7. Nasr I, McGrath EJ, Harwood CA, et al. British Association of Dermatologists guidelines for the management of adults with basal cell carcinoma 2021. Br J Dermatol. 2021 Nov;185(5):899–920.

8. Ali SR, Dobbs TD, Jovic M, et al. Improving the effectiveness of multidisciplinary team meetings in skin cancer: Analysis of national Cancer Research UK survey responses. J Plast Reconstr Aesthet Surg. 2023 (In Press)

9. NHS England and NHS Improvement. Streamlining multi-disciplinary team meetings: guidance for cancer alliances. 2020. Available from: https://www.england.nhs.uk/wp-content/uploads/2020/01/multi-disciplinary-team-streamlining-guidance.pdf (last accessed 3 April 2023)

10. Ali SR, Dobbs TD, Mohamedbhai H, et al. Evaluating remote skin cancer multidisciplinary team meetings in the United Kingdom post-COVID-19. J Plast Reconstr Aesthet Surg. 2023 (In Press)

11. Harrison CJ, Sidey-Gibbons CJ. Machine learning in medicine: a practical introduction to natural language processing. BMC Med Res Methodol. 2021 Jul 31;21(1):158.

12. Mellia JA, Basta MN, Toyoda Y, et al. Natural Language Processing in Surgery: A Systematic Review and Meta-analysis. Ann Surg. 2021 May 1;273(5):900–8.

13. Ali SR, Strafford H, Dobbs TD, et al. Development and validation of an automated basal cell carcinoma histopathology information extraction system using natural language processing. Front Surg. 2022 Aug 24;9:870494.

14. Ali SR, Dobbs TD, Jovic M, et al. Validating a novel natural language processing pathway for automated quality assurance in surgical oncology: incomplete excision rates of 34 955 basal cell carcinomas. Br J Surg. 2023 Mar 20;znad055.

15. Abide JM, Nahai F, Bennett RG. The meaning of surgical margins. Plast Reconstr Surg. 1984 Mar;73(3):492–7.

16. NHS Digital. SNOMED CT. Available from: https://digital.nhs.uk/services/terminology-and-classifications/snomed-ct (last accessed 3 April 2023)

17. Slater D, Barrett P. Standards and datasets for reporting cancers: Dataset for histopathological reporting of primary basal cell carcinoma. 2019. Available from: https://www.rcpath.org/uploads/assets/53688094-791e-4aaa-82cec42c3cb65e35/Dataset-for-histopathological-reporting-of-primary-cutaneous-basal-cell-carcinoma.pdf (last accessed 3 April 2023)

18. Vaughan D. Multiclass averaging. Available from: https://yardstick.tidymodels.org/articles/multiclass.html?q=micro%20a (last accessed 3 April 2023)

19. Topol E. The Topol Review: Preparing the healthcare workforce to deliver the digital future. Available from: https://topol.hee.nhs.uk/wp-content/uploads/HEE-Topol-Review-2019.pdf (last accessed 3 April 2023)

20. McKinney SM, Sieniek M, Godbole V, et al. International evaluation of an AI system for breast cancer screening. Nature. 2020 Oct;586(7829):E19.

21. National Institute for Health and Care Excellence. Artificial intelligence in mmammmmography. Available from: https://www.nice.org.uk/advice/mib242/resources/artificial-intelligence-in-mammography-pdf-2285965629587653 (last accessed 3 April 2023)

22. Public Health England. NHS breast screening programme (BSP). Available from: https://www.gov.uk/guidance/breast-screening-programme-overview (last accessed 3 April 2023)

23. Ghassemi M, Oakden-Rayner L, Beam AL. The false hope of current approaches to explainable artificial intelligence in health care. Lancet Digit Health. 2021 Nov;3(11):e745–50.

24. Vaswani A, Shazeer N, Parmar N, et al. Attention is all you need. Adv Neural Inf Process Syst. 2017;30.

25. Andrew TW, Hamnett N, Roy I, et al. Machine-learning algorithm to predict multidisciplinary team treatment recommendations in the management of basal cell carcinoma. Br J Cancer. 2022 Mar;126(4):562–8.

26. Barrett PD, Barrett HE. An audit into use of minimum dataset reporting of skin cancers in the North of England Cancer Network. Pathogenesis. 2015 Jan 1;2(1):5–8.

27. Legislation.gov.uk. The Medical Devices Regulations 2002 [Internet]. Available from: https://www.legislation.gov.uk/uksi/2002/618/contents (last accessed 7 July 2023)

28. Digital Regulations Innovation. Understanding regulations for medical devices under the UK MDR 2002 [Internet]. Available from: https://www.digitalregulations.innovation.nhs.uk/developers-guidance/all-developers-guidance/uk-mdr-2002-understanding-regulations-medical-devices/ (last accessed 7 July 2023)

29. Digital Regulations Innovation. Understanding regulations for medical devices under the UK MDR 2002 [Internet]. Available from: https://www.digitalregulations.innovation.nhs.uk/developers-guidance/all-developers-guidance/uk-mdr-2002-understanding-regulations-medical-devices/ (last accessed 7 July 2023)
